# Use of Vanadium Complexes Bearing Naphthalene-Bridged Nitrogen-Sulfonate Ligands as Catalysts for Copolymerization of Ethylene and Propylene

**DOI:** 10.3390/polym9080325

**Published:** 2017-07-31

**Authors:** Xiufeng Hao, Chundi Zhang, Lin Li, Hexin Zhang, Yanming Hu, Daifeng Hao, Xuequan Zhang

**Affiliations:** 1 College of Chemistry, Jilin University, Changchun 130012, China; kuailezhangchundi@126.com (C.Z.); lynn_li@smics.com (L.L.); 2 Key Laboratory of Synthetic Rubber, Changchun Institute of Applied Chemistry, Chinese Academy of Sciences, Changchun 130022, China; polyhx@ciac.jl.cn (H.Z.); ymhu@ciac.ac.cn (Y.H.); 3 Department of Wound Repair Center, Burn & Plastic Surgery, First Affiliated hospital of Chinese PLA General Hospital, Beijing 100037, China; hdf304@126.com

**Keywords:** vanadium catalyst, ethylene/propylene copolymerization, polyolefin

## Abstract

Vanadium complexes bearing naphthalene-bridged nitrogen-sulfonate ligand ([ê^2^(N,O)-8-(PhN)-1-naphthalenesulfonato]VOCl (**1a**) and [ê^2^(N,O)-8-(PhN)-1-naphthalenesulfonato]VCl_2_ (**1b**)) were synthesized. Activated by ethylaluminium sesquichloride (EASC) and in the presence of ethyl trichloroacetate (ETCA) as reactivator, complexes **1a** and **1b** showed activities of up to 39.1 kg polymer (mol V)^−1^ h^−1^, affording the copolymers with high molecular weights (*M*_w_ up to 28 × 10^4^) and narrow molecular weight distributions (*M*_w_/*M*_n_ ~ 3.0) as well as high propylene incorporation of up to 49.4%. Compared to the traditional VOCl_3_ system, these complexes exhibited higher propylene incorporation ability and higher catalytic activities especially at high polymerization temperatures of 50 °C and above. Determined by DSC and ^13^C NMR, the copolymers obtained with **1a** and **1b** had more random structures than that with the VOCl_3_ system.

## 1. Introduction

Design and synthesis of transition metal-based catalysts for olefin polymerization is a long-standing research subject since the pioneering work by Ziegler and Natta, and some of them have been successfully utilized in industrial applications [1,2,3,4,5,6,7]. Vanadium-based catalysts, though less active than those of other systems, exhibit interesting features in olefin (co)polymerization process [8,9,10,11,12,13]. Such catalysts can produce high molecular weight polymers with narrow molecular weight distributions, ethylene/á-olefin copolymers having high á-olefin incorporation, and syndiotactic polypropylene. Particularly, vanadium-based catalysts are widely used worldwide for the industrial production of ethylene/propylene copolymers and ethylene/propylene/diene terpolymers, a rapid growing class of elastomers. However, despite the unique characteristics and practical application of the classical Ziegler-type vanadium catalysts, they suffered from the decay of activity during the polymerization process as a result of reduction of active species to less active or even inactive low-valent vanadium species, which is more prevalent at elevated temperatures. Therefore, the development of vanadium catalysts with high activity and stability has been recognized as an attractive target.

Two strategies are known to improve the catalytic performance of vanadium catalysts. The use of reoxidants such as ethyl trichloroacetate and chlorinated hydrocarbons (so-called “rejuvenators” or “promoters” in the literature) is well-known for maintaining the higher (active) oxidation state of vanadium systems by the reactivation of vanadium (II) center to vanadium (III) species [14,15]. The incorporation of appropriate ancillary ligands modification vanadium-based systems has proved another effective way for stabilizing the catalytically active species [8,9,10,11,12,13,16,17,18,19,20,21,22,23,24,25,26,27,28,29,30,31,32,33,34,35]. For instance, Nomura and coworkers reported that imido-based vanadium complexes containing phenoxyimine or anilidomethylpyridine coligands are efficient catalyst precursors for olefin (co)polymerization [19,20,21]. Redshaw et al. prepared a variety of vanadium complexes bearing chelating aryloxides, which exhibit high activity in ethylene polymerization even at elevated temperatures [10,23,24]. Li et al. reported various [N, O], [N, N], and [O, P=O] ligands such as β-enaminoketonato, iminopyrrolide, and phenoxy-phosphine oxide supported vanadium complexes with high activity and comonomer incorporation ability in ethylene (co)polymerization [9,25,26,27]. Abernethy reported a highly stable *N*-heterocyclic carbene vanadium (V) trichloride oxide complex even in air, but its catalysis properties was not investigated [28]. Nomura synthesized a series of (imido) vanadium (V) complexes bearing anionic *N*-heterocyclic carbenes, which exhibit remarkable catalytic activity for ethylene polymerization in the presence of Al(*i*-Bu)_3_ [29]. Recently, chelating sulfonated ligands have attracted considerable interest in the design of highly active catalysts because of their remarkable electronic asymmetry as well as geometric flexibility [36,37,38]. Given the impressive activities associated with chelating sulfonated ligand-based Ru, Pd, and Ni catalytic chemistry including olefin metathesis, olefin polymerization, and copolymerization with polar vinyl monomers [39,40,41], such hybrid ligands would be attractive candidates for preparation of vanadium catalysts with high performance. As mentioned above, many efforts have been made to develop novel vanadium catalysts, and a variety of vanadium complexes with high activities and incorporation ability have been successfully prepared. VOCl_3_ has been widely used for the industrial production of ethylene/propylene copolymers and ethylene/propylene/diene terpolymers. Therefore, in the present study, two novel vanadium complexes bearing naphthalene-bridged nitrogen-sulfonate ligand, [ê^2^(N,O)-8-(PhN)-1-naphthalenesulfonato]VOCl and [ê^2^(N,O)-8-(PhN)-1-naphthalenesulfonato]VCl_2_, were synthesized, and due to the ease of preparation, these complexes might be promising candidates for practical applications. Their catalytic behaviors toward ethylene/propylene copolymerization were investigated by comparing with the results of the traditional VOCl_3_-based catalyst as a control system. Furthermore, the effects of cocatalyst quantity, polymerization temperature, and time on the polymerization were examined in detail.

## 2. Experimental Section

### 2.1. General Considerations and Materials

All the manipulations were carried out under nitrogen atmosphere using standard Schlenk techniques. Hexane and tetrahydrofuran were refluxed over sodium/diphenyl ketyl under nitrogen and then distilled prior to use. Vanadium (V) trichloride oxide (VOCl_3_) and vanadium tetrachloride (VCl_4_) were purchased from Nanjing XingHong Chemical Co (Nanjing, China) and used as received. 8-(Phenylamino)naphthalene-1-sulfonic acid and ethyl trichloroacetate (ETCA) were purchased from Aladdin Co (Shanghai, China). Ethylaluminium sesquichloride (EASC) was purchased from Sigma-Aldrich Co (Saint Louis, MO, USA) and diluted to 1.0 mol/L solution by hexane. Polymerization grade ethylene and propylene were further purified by passing through columns of 5 Å molecular sieves and MnO. Other chemicals were commercially available and used without further purification.

The NMR spectra of polymers were recorded on a Bruker 300 MHz spectrometer at 135 °C with 1,2-dichlorobenzene-d4 as a solvent. Elemental analysis was conducted with Carlo Erba 1106 and ST02 apparatus. Differential scanning calorimetry (DSC) measurements were performed with a TA instrument DSC 2920 at a heating rate of 10 °C/min. The molecular weight and molecular weight distribution of the polymers were determined by gel permeation chromatography (GPC) at 140 °C on a PL-GPC 220 high temperature chromatograph equipped with three PL gel 10 mm Mixed-B LS type columns. 1,2,4-Trichlorobenzene was employed as eluant at a flow rate of 1.0 mL/min. The calibration was made by polystyrene standard (Easi-Cal PS-1 PL Ltd.).

### 2.2. Synthesis of Complexes

#### 2.2.1. Synthesis of Complex **1a**

A 100 mL three-necked flask was equipped with a three-way stopcock and a magnetic stirring bar. After the flask was flushed with nitrogen, tetrahydrofuran (40 mL) and 8-(phenylamino) naphthalene-1-sulfonic acid (1.5 g, 5.0 mmol) was added, and then vanadium (V) trichloride oxide (0.88 g, 5.1 mmol) was added at −20 °C. The reaction mixture was warmed to room temperature and stirred for 48 h. The solvent was removed in vacuo, the resulting solid was repeatedly washed with hexane and dried under vacuum to give a brown solid; yield (1.2 g, 3.0 mmol), 60%. IR (KBr, cm^−1^): 1618, 1602, 1483, 1251, 1141, 1037, 999, 762, 747, 681. ^1^H NMR (CDCl3) δ (ppm): 8.28 (s, 1H, Ar), 7.82 (s, 1H, Ar), 7.61−7.12 (m, 8H, Ar), 6.78 (s, 1H, Ar). Anal. Calcd for C16H11ClNO4SV: C, 48.08; H, 2.77; N, 3.50; S, 8.02. Found: C, 50.80; H, 2.56; N, 2.99; S, 8.50.

#### 2.2.2. Synthesis of Complex **1b**

A 100 mL three-necked flask was equipped with a three-way stopcock and a magnetic stirring bar. After the flask was flushed with nitrogen, tetrahydrofuran (40 mL) and 8-(phenylamino) naphthalene-1-sulfonic acid (1.5 g, 5.0 mmol) was added, and then vanadium (IV) tetrachloride (0.98 g, 5.1 mmol) was added at −20 °C. The reaction mixture was warmed to room temperature and stirred for 48 h. The solvent was removed in vacuo, the resulting solid was repeatedly washed with hexane and dried under vacuum to give a brown solid; yield 58%, IR (KBr, cm^−1^): 1618, 1604, 1479, 1247, 1199, 1137, 1033, 1003, 740, 680. Anal. Calcd for C_16_H_11_Cl_2_NO_3_SV: C, 45.84; H, 2.65; N, 3.34; S, 7.65. Found: C, 46.50; H, 2.01; N, 2.96; S, 8.11.

### 2.3. General Procedure for Copolymerization of Ethylene and Propylene

Copolymerization was carried out in a glass reactor with vigorous stirring. A typical polymerization procedure is described as following. After the reactor was repeatedly evacuated and flushed with nitrogen, hexane (100 mL) was added, and premixed ethylene/propylene gas (1:2 mole ratio) was then introduced until saturation. Copolymerization was initiated by the sequential addition of certain amounts of EASC, vanadium complex, and ETCA and carried out at 1 atm of mixture gas pressure and a desired temperature. The polymerization was quenched by addition of excessive methanol containing a small amount of hydrochloric acid, and the precipitated polymer was filtered, repeatedly washed with methanol, and dried under vacuum at 40 °C to constant weight.

## 3. Results and Discussion

### 3.1. Synthesis of Vanadium Complexes

**Scheme 1:**
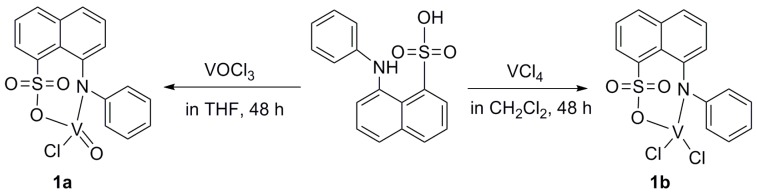
Synthesis of vanadium complexes **1a** and **1b**.

The synthetic route for the vanadium complexes is shown in Scheme 1. The reaction of VCl_4_ or VOCl_3_ with 1.0 equivalent of 8-(phenylamino)naphthalene-1-sulfonic acid in THF afforded complexes **1a** and **1b** in moderate yields (60% and 58%, respectively). While the attempt to obtain the crystals of these two complexes was failed, these complexes were identified by ^1^H NMR (Figure S1) and element analysis. The signal at 11.2 ppm assignable to N*H* group of the ligand disappeared in the H^1^ NMR spectrum of complex, and other signals meanwhile remained intact. This result combined with the elemental analysis data indicates the formation of complex. These two complexes are relatively stable in air and soluble in common organic solvents such as CHCl_3_, CH_2_Cl_2_, and toluene.

### 3.2. Ethylene/Propylene Copolymerization

**Figure 1:**
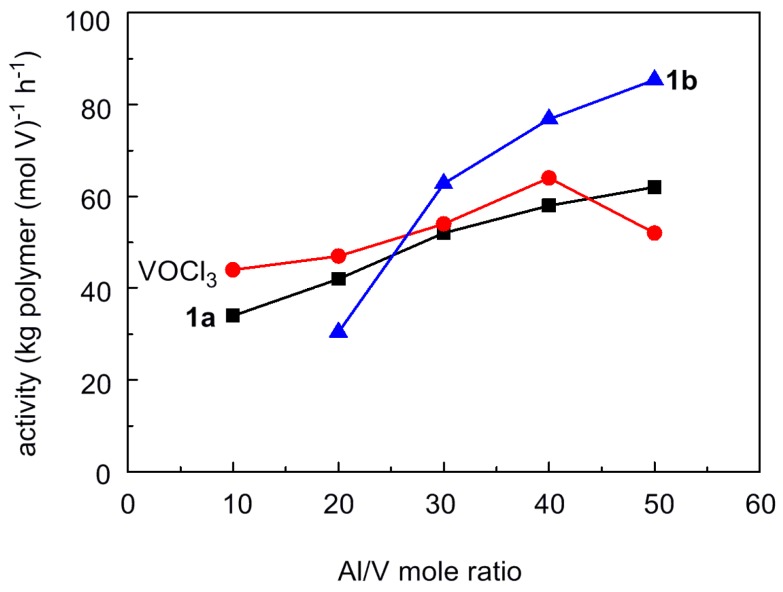
Effect of Al/V ratio on on activity of vanadium-based catalysts in ethylene/propylene polymerization. Reaction conditions: [V] = 0.5 mmol/L, [ETAC]/[V] = 10, hexane 100 mL, 30 °C, 30 min, 1 bar of ethylene/propylene (1/2, molar ratio) pressure.

It is known that vanadium compounds could be reduced by alkylaluminum to different oxidation state, V(IV), V(III), and V(II), depending on the Al/V ratio [8], and in turn, the dosage of cocatalyst remarkably influenced the activity of vanadium catalysts. In order to investigate the effect of Al/V mole ratio on the catalytic behavior, the ethylene/propylene copolymerization was conducted with complexes **1a** and **1b** in the presence of the cocatalyst EASC and the reactivator ETCA along with commercially used VOCl_3_ for comparison. As shown in Figure 1, in all cases, the catalytic activity increased as the Al/V mole ratio increased from 10 to 40, and the activity of VOCl_3_-based system was higher than that of **1a**-system whilst somewhat lower than the **1b**-system. However, a further increased Al/V ratio to 50 resulted in a decrease of activity of VOCl_3_ due to the reduction of the active species by the cocatalyst, whereas the **1a** and **1b** catalyst system exhibited an increase in activities. These results indicate that the naphthalene-bridged nitrogen-sulfonate ligand protects **1a** and **1b** catalysts against catalyst deactivation typically through reduction by an aluminum cocatalyst to the inactive divalent vanadium species. Since the highest activity of traditional VOCl_3_-based system was obtained at an Al/V mole ratio of 40, it was used in the further experiments to compare the catalytic performance between the newly synthesized vanadium complexes and VOCl_3_.

In the cases of vanadium-catalyzed olefin polymerization, it is well established that the activity can be enhanced in the presence of reactivator ETCA, which reactivates the low-valent, less active or inactive V(II) species to active vanadium (III) species [15,42]. As shown in Table 1, in all cases, the activities of complexes **1a**, **1b**, and VOCl_3_ in the presence of ETCA (38.4~78.4 kg polymer (mol V)^−1^ h^−1^ bar^−1^) were much higher than those of ETCA-free systems (18.6~41.2 kg polymer (mol V)^−1^ h^−1^ bar^−1^). Similar results were observed in other vanadium catalyst systems [15,43]. Especially, in the absence of ETCA, the activities of complexes **1a** and **1b** were more or less than that of VOCl_3_, further suggesting that the nitrogen-sulfonate ligand protects active species generated by **1a** and **1b** from deactivation. The highest activity was observed at ETCA/V ratio of 10 in all cases, and further increased the level of ETCA resulted in a slightly decreased activity. Among three systems, **1b** gave the highest catalytic activity of 78.4 kg polymer (mol V)^−1^ h^−1^ bar^−1^, which is higher than those of **1a** and VOCl_3_ (52.8 and 64.0 kg polymer (mol V)^−1^ h^−1^ bar^−1^, respectively). Although the detailed reason for this observation is not clear yet, it is speculated that the role played by ETCA to regenerate active vanadium species is more pronounced in the case of **1b**, in which the presence of ETCA led to ca. a 4-fold increase in activity, much higher than that in the cases of **1a** and VOCl_3_. On the other hand, the molecular weight of the resulting copolymers decreased with the increase in the ETCA/V ratio. This result might be due to the higher concentration of active species generated in the presence of the reactivator. It is worth noting that the molecular weights of the resulting copolymers obtained with complexes **1a** and **1b** were higher than that with commercially used VOCl_3_. Whereas VOCl_3_ system produced copolymers with rather broad molecular weight distributions (*M*_w_/*M*_n_ = 5.46–7.69), the unimodal GPC curves and the narrow molecular weight distributions suggested that the formation of single-site active species in **1a** and **1b** systems. These facts indicate that EASC does not abstract the naphthalene-bridged nitrogen-sulfonate ligand from vanadium center to the aluminum cocatalyst, as that proposed in the cases of tridentate (OSO) ligand-supported vanadium complexes [44]. Meanwhile, the presence of ETCA also influenced the propylene incorporation in the resulting copolymers. The increase in ETCA/V ratio led to an enhancement in propylene incorporation. For instance, the incorporation of propylene in the copolymers obtained with **1b** was 49.4% at ETCA/V ratio of 20, which is much higher than that of in the absence of ETCA (33.1%). Noticeably, under identical conditions the incorporation of propylene in the cases of **1a** and **1b** was higher than that of VOCl_3_.

**Figure 2:**
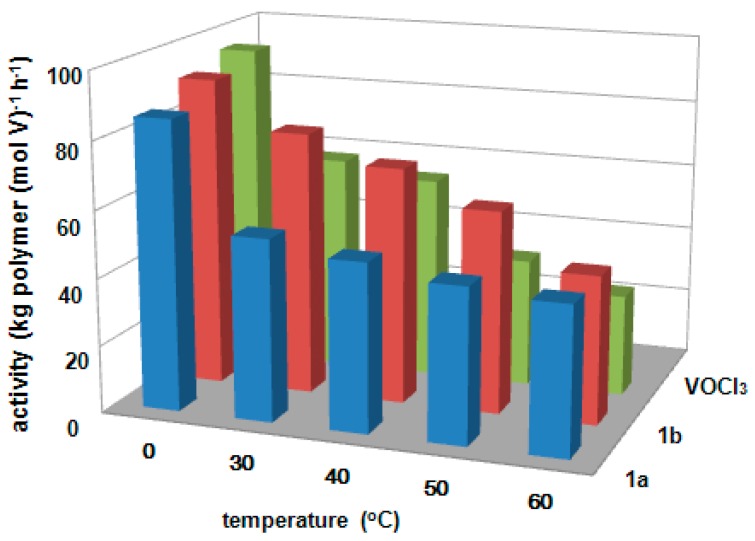
Effect of polymerization temperature on activity of vanadium-based catalysts in ethylene/propylene polymerization.

The influence of polymerization temperature on the activity of these systems was further investigated, and the results are listed in Figure 2. The catalytic activities of complexes **1a** and **1b** as well as VOCl_3_ decreased with an increase in temperature as commonly observed in other vanadium catalysts, and the reason might be that the increase of polymerization temperature resulted in the accelerated reduction of active high valent vanadium species. Complexes **1a** and **1b** exhibited higher activities at high temperatures of 50 °C and above compared with VOCl_3_, suggesting the potential high thermal stability of the two complexes, although they showed more or less lower catalytic activities than that of VOCl_3_ at 0 °C. The fact of maintaining high activity at high temperatures is important from the viewpoint of practical application.

**Figure 3:**
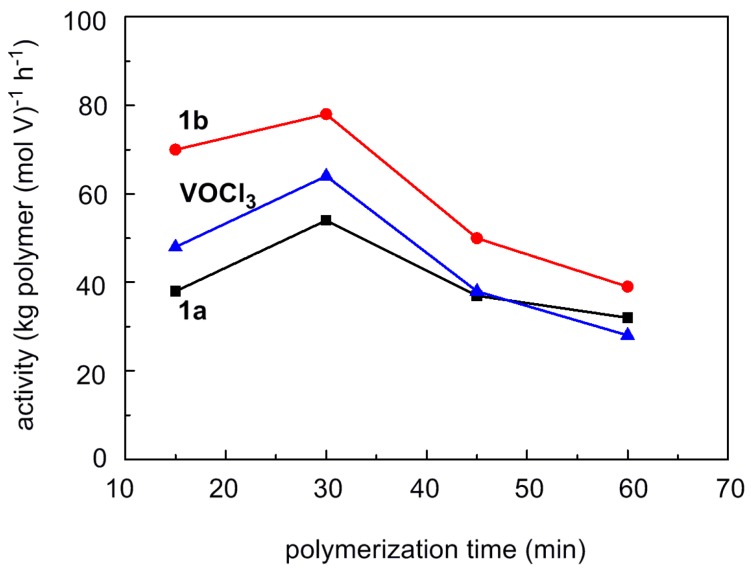
Effect of polymerization time on activity of vanadium-based catalysts in ethylene/propylene polymerization.

Catalyst lifetime studies were carried out with complexes **1a** and **1b**, as well as VOCl_3_-based systems at 30 °C, and the results are shown in Figure 3. The three catalysts displayed similar trends in catalytic performance with respect to the activity against polymerization time. In all cases, the catalytic activity increased at the first stage and was found to peak at ca. 30 min, 53, 78, and 64 kg polymer (mol V)^−1^ h^−1^ for **1a**, **1b**, and VOCl_3_, respectively, whereas further prolonging polymerization time resulted in a greater or lesser decrease in activity.

### 3.3. Polymer Characterization

**Figure 4:**
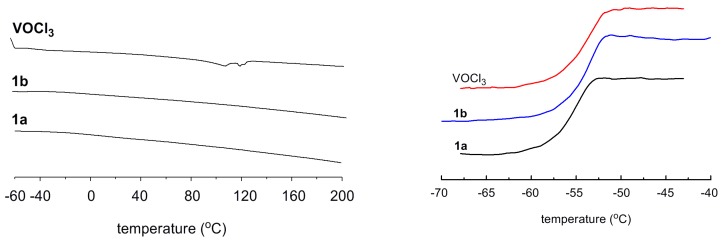
DSC curves of ethylene/propylene copolymers obtained with vanadium-based catalysts (**left**, VOCl_3_, Run 3; **1a**, Run 6; **1b**, Run 10 in Table 1) and partly enlarged curves (**right**).

The thermal behaviors of the copolymer samples were similar. As shown in Figure 4, the DSC thermograms of the copolymers exhibit only a glass transition temperature. The *T*_g_s of the copolymers obtained with **1a** and **1b** are −56.9 °C and −56.5 °C, respectively, which are slightly lower than that with VOCl_3_ (−55.9 °C). The copolymers obtained with **1a** and **1b** did not display melting points, whereas the ethylene-propylene copolymer obtained with VOCl_3_ had weak melting endotherms at 100 to 120 °C, although the X-ray spectrum indicates that the polymer is amorphous (Figure S2). These results might be due to the more randomly distributed monomer units and/or the increasing propylene incorporation amount in the copolymers than that obtained with VOCl_3_ system.

**Figure 5:**
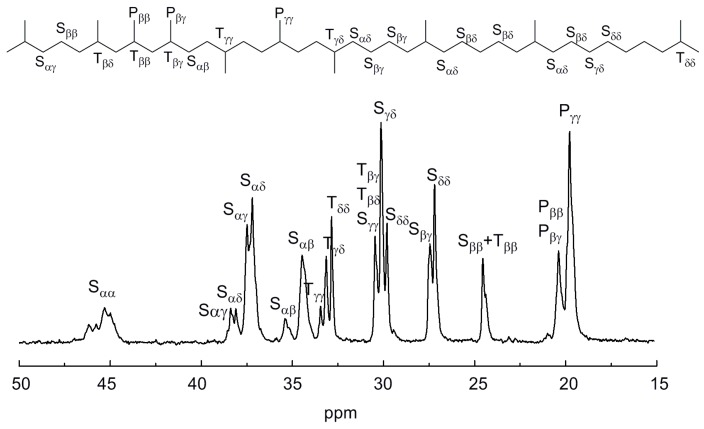
^13^C NMR spectrum of ethylene/propylene copolymer obtained with **1a**/EASC catalyst (sample from Run 3 in Table 1).

**Figure 6:**
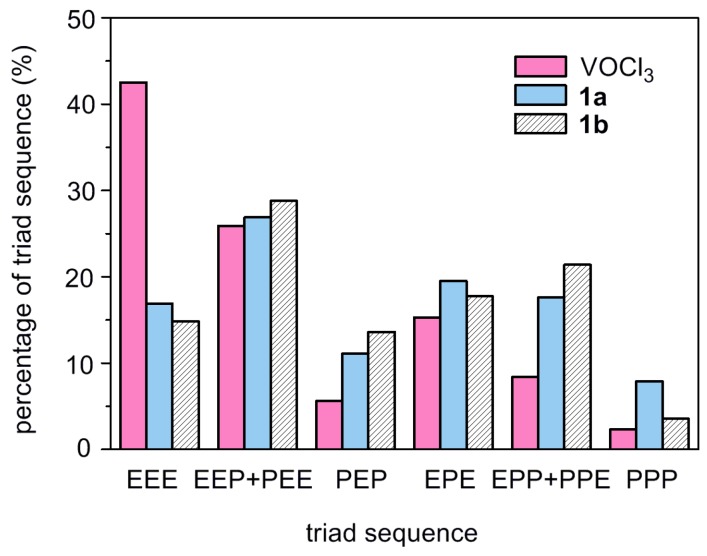
Monomer sequence distribution of ethylene/propylene copolymers determined from ^13^C NMR spectra (VOCl_3_, Run 3; **1a**, Run 6; **1b**, Run 10 in Table 1).

Since the sequence distribution is one of the important factors determining the mechanical properties of ethylene/propylene copolymer, the microstructures of ethylene/propylene copolymers were further characterized by ^13^C NMR. Figure 5 shows the typical ^13^C NMR spectrum of the resulting copolymer, and the peaks are assigned according to the literature [45,46,47,48]. The composition of the copolymers was estimated from the ^13^C NMR spectra according to the calculation method proposed by Randall [49], and the results are shown in Figure 6. The copolymer obtained with VOCl_3_ possessed higher content of EEE triads than those with **1a** and **1b**, while the content of EPE triad is lower, suggesting that in the cases of **1a** and **1b** (Figure S3) propylene inserted more randomly along the polymer chain. On the other hand, compared to VOCl_3_ (Figure S4), **1a** and **1b** afforded polymers with more or less higher PPP triad contents, which might be due to the higher propylene incorporation.

## 4. Conclusions

In summary, we have synthesized two novel naphthalene-bridged nitrogen-sulfonate ligand-supported vanadium complexes. The complexes exhibited high catalytic activities in ethylene/propylene copolymerization in the presence of EASC as a cocatalyst and ETCA as a reactivator, affording with high molecular weight copolymers. The naphthalene-bridged nitrogen-sulfonate ligand protects catalysts against catalyst deactivation typically through reduction by an aluminum cocatalyst to the inactive divalent vanadium species. Compared to the traditional VOCl_3_ system, the newly synthesized vanadium complex-based catalysts showed higher activities at high polymerization temperatures. The unimodal GPC curves and narrow molecular distributions of the resulting polymers suggested the presence of single active species in the polymerization systems. Meanwhile, the complexes displayed higher propylene incorporation ability, and the resulting copolymers possessed more random structures. Taking the advantages of facile preparation, high activity especially at elevated temperatures and propylene incorporation ability as well as high molecular weight and uniform composition of the resulting polymers, these vanadium complexes are promising as practically useful catalysts for ethylene/propylene copolymerization.

## Figures and Tables

**Table 1:** Ethylene/propylene copolymerization with V complex/Et_3_Al_2_Cl_3_^a^.

Run	Cat.	ETCA/V	Yield (g)	Activity ^b^	*M*_w_ × 10^−4 c^	*M*_w_/*M*_n_^c^	C_3_ Incorp. (mol%) ^d^
1	1a	0	1.03	41.2	23.1	3.16	36.0
2	1a	5	1.30	52.0	12.7	2.09	39.0
3	1a	10	1.32	52.8	8.3	3.17	40.6
4	1a	20	1.22	48.8	4.0	2.66	48.2
5	1b	0	0.50	20.0	28.1	3.01	33.1
6	1b	10	1.96	78.4	19.6	2.63	37.3
7	1b	20	1.55	62.0	6.1	2.77	49.4
8	VOCl_3_	0	0.93	18.6	19.5	5.69	25.1
9	VOCl_3_	5	0.96	38.4	10.3	7.25	28.9
10	VOCl_3_	10	1.60	64.0	6.4	7.69	33.0
11	VOCl_3_	20	1.53	61.2	2.2	5.46	40.2

^a^ Polymerization conditions: 100 mL of hexane, 30 °C, 30 min; catalyst, 50 μmol; Al/V = 40; propylene/ethylene (2/1, mole ratio) pressure, 1 atm; ETCA, ethyl trichloroacetate. ^b^ kg polymer (mol V)^−1^ h^−1^. ^c^ Determined by GPC. ^d^ Determined by ^13^C NMR.

## Supplementary Materials

The following are available online at www.mdpi.com/2073-4360/9/8/325/s1, Figure S1: ^1^H NMR spectra of the ligand and complex **1a**. Figure S2: XRD spectrum of ethylene/propylene copolymer obtained with 1**a**/EASC catalyst (sample from run 3 in Table 1). Figure S3: ^13^C NMR spectrum of ethylene/propylene copolymer obtained with 1**b**/EASC catalyst (sample from run 6 in Table 1). Figure S4: ^13^C NMR spectrum of ethylene/propylene copolymer obtained with VOCl_3_/EASC catalyst (sample from run 10 in Table 1).
