# Supplementary material for: Use of Vanadium Complexes Bearing Naphthalene-Bridged Nitrogen-Sulfonate Ligands as Catalysts for Copolymerization of Ethylene and Propylene
Source: Polymers (Basel). 2017 Jul 31;9(8):325. doi: 10.3390/polym9080325 (PMC6418982; doi:10.3390/polym9080325)
Supplement: Supplementary file 1 [file polymers-09-00325-s001.pdf]

Supporting Information

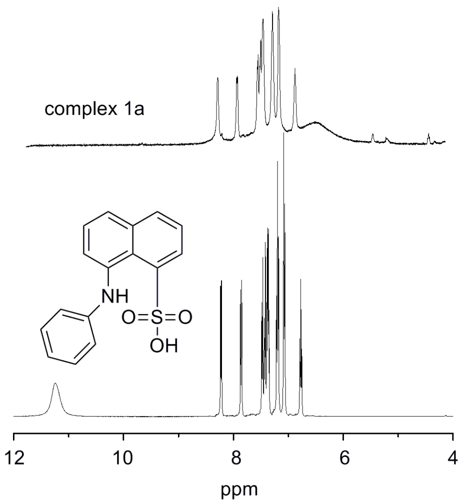

Figure S1. ^{1}H NMR spectra of the ligand and complex **1a**.

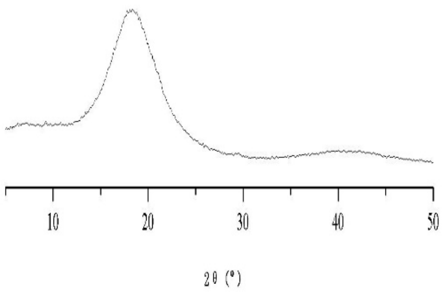

Figure S2. XRD spectrum of ethylene/propylene copolymer obtained with 1**a**/EASC catalyst (sample from run 3 in Table 1).

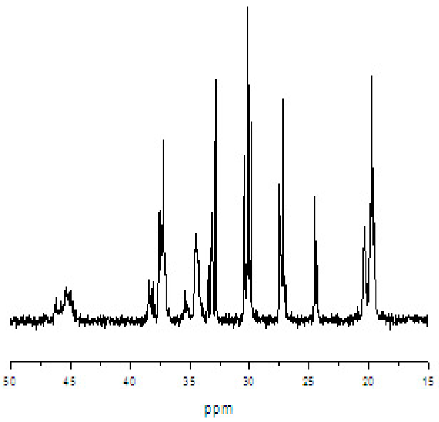

Figure S3. ^{13}C NMR spectrum of ethylene/propylene copolymer obtained with 1**b**/EASC catalyst (sample from run 6 in Table 1).

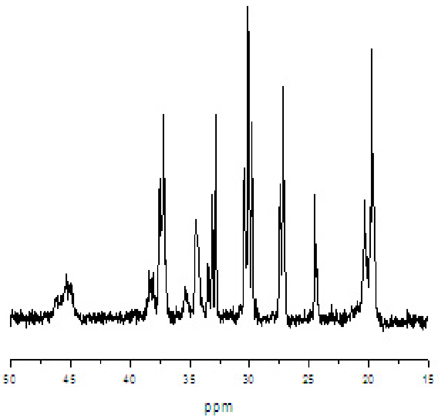

Figure S4. ^{13}C NMR spectrum of ethylene/propylene copolymer obtained with $VOCl_3$/EASC catalyst (sample from run 10 in Table 1).
